# Nuestras Historias- Designing a novel digital story intervention through participatory methods to improve maternal and child health in the Peruvian Amazon

**DOI:** 10.1371/journal.pone.0205673

**Published:** 2018-11-05

**Authors:** Neha P. Limaye, Andrea C. Rivas-Nieto, Cesar P. Carcamo, Magaly M. Blas

**Affiliations:** 1 Department of Internal Medicine-Pediatrics, Harvard Brigham and Women’s Hospital, Boston, Massachusetts, United States of America; 2 Boston Children’s Hospital, Boston, Massachusetts, United States of America; 3 Boston Medical Center, Boston, Massachusetts, United States of America; 4 Department of Epidemiology, STD and HIV Unit, School of Public Health and Administration, Universidad Peruana Cayetano Heredia, Lima, Peru; Aga Khan University, KENYA

## Abstract

**Background:**

In rural areas of the Loreto region within the Peruvian Amazon, maternal mortality rate is above the national average and the majority of women deliver at home without care from a trained health care provider.

**Methods:**

To develop community-tailored videos that could be used for future interventions, we conducted Photovoice and digital storytelling workshops with community health workers (CHW) and mothers from 13 rural communities in the Parinari district. Through Photovoice we recognized local barriers to healthy pregnancies. Participants (n = 28) were trained in basic photography skills and ethics. They captured photos representing perceived pregnancy-related road-blocks and supports, and these photos identified central themes. Participants recorded personal stories and “storyboarded” to develop digital stories around these themes, and a Digital Story Curriculum called Nuestras Historias (Our Stories), was created. An acceptability survey of the digital stories was then conducted including 47 men (M) and 60 women (F).

**Results:**

According to the PhotoVoice workshops, pregnancy-related problems included: lack of partner support, domestic violence, early pregnancies, difficulty attending prenatal appointments, and complications during pregnancy and delivery. Over 30 stories on these themes were recorded. Seven were selected based on clarity, thematic relevance, and narrative quality and were edited by a professional filmmaker. The acceptability survey showed that local participants found the digital stories novel (M = 89.4%, F = 83.3%), relatable (M = 89.4%, F = 93.2%), educational (M = 91.5%, F = 93.3%) and shareable (M = 100%, F = 100%). Over 90% of respondents rated the digital stories as “Excellent” or “Good”, found the videos “Useful” and considered them “Relevant” to their communities.

**Conclusions:**

The digital stories address community-specific problems through narrative persuasion using local voices and photography. This combination had a high acceptability among the target population and can serve as a model for developing educational strategies in a community-tailored manner. This package of seven videos will be further evaluated through a cluster randomized trial.

## Introduction

As part of a global effort to improve maternal and child health (MCH), the Sustainable Development Goals sets a target for nations to reach a maternal mortality rate (MMR) of 70 or fewer deaths per 100,000 live births. In Peru, the estimated MMR is 93 deaths per 100,000 live births [1] but may be even higher given the significant under-registration of maternal deaths [2]. To address this, the government has implemented programs to increase the presence of skilled birth attendants and the percent of women getting adequate prenatal care, which have greatly improved maternal and child health indicators. However, some of Peru’s rural regions lag severely behind in these improvements [3].

Loreto is Peru’s largest region and contains most of the Peruvian Amazon, where rural villages are mainly bound by the river [4]. Maternal mortality in Loreto is significantly above the national average [5] and hemorrhage remains the leading cause of death [6]. The neonatal mortality rate is estimated to be 31.4 per 1000 live births and the perinatal mortality rate is close to 49.7 per 1000 pregnancies, with infection as the main cause of death (43%) [2]. Despite efforts to promote access to health services, 80.2% of women in rural riverine areas deliver at home rather than travel to find a health center [7]. This is due to a large variety of factors, including transportation difficulties, cultural preferences, and understaffed and under resourced health centers [8].

In such a remote and resource-limited setting as Loreto, mobile technology can be the answer to reaching the population and addressing MCH disparities [9,10]. Most MCH mobile health promotion programs use text messages or voice recordings to provide health information to women [11]. However, information alone is not enough to produce behavior change; motivation is also required to actually inspire behavior change [12]. One growing strategy in behavior change communication is using videos to provide information and motivation, especially in populations with lower literacy and areas where not every family has access to a cell phone. Studies in other health fields have shown that educational videos can be effective in motivating subjects and enabling long-term behavioral change [13]. The few existing studies that have measured the effect of educational videos on pregnant women show that they can change attitudes and behaviors related to prenatal care [14–16].

While some basic topics in prenatal education are applicable across many populations, it is important to note that the communities of Loreto have their own specific geographic and socio-cultural barriers to MCH. Designing an effective MCH video intervention for Loreto thus requires understanding these specific barriers and addressing them directly. Acknowledging that the community members themselves are the most qualified to explain and address these barriers, with the deepest knowledge of their local issues, this study uses the principles of community-based participatory research (CBPR) in order to create a novel video intervention.

CBPR promotes involvement of the community, inviting them to share their knowledge, needs, and experiences, and participate in intervention design [17,18]. One emerging CBPR method is PhotoVoice, which uses photography to promote empowerment in the community. It has three main objectives: “*1) To enable people to record and reflect their community strengths and concerns*, *2) to promote critical dialogue and knowledge about important community issues through large and small group discussion of photographs*, *and 3) to reach policymakers”* [19]. PhotoVoice has been successfully applied in maternal health projects involving vulnerable populations in low- and middle income countries, showing promising results in terms of increasing knowledge and affecting policy change [20,21]. This study uses PhotoVoice to conduct a needs assessment and better understand issues surrounding pregnancy in the Parinari district of Loreto.

Focusing on the issues uncovered through PhotoVoice, the study works with community members to develop a Digital Story Curriculum (DSC). Digital stories are short, 3–5 minute videos that use audio-recorded personal narratives accompanied by narrator-selected photos, and have developed recent popularity as a public health tool [22]. Digital stories are made by community members and are thus inherently shaped around specific community needs. They use the recognized power of narrative persuasion coming from a relatable narrator in order to bring about mental shifts and behavioral changes [23]. There are no known studies on the effect of digital stories on MCH, but given the success of digital stories in other public health arenas and the need for new forms of behavior change communication in maternal health, their potential is worth exploring [23]. The study concludes by piloting the DSC with community members and surveying to gauge initial responses to the stories and acceptability.

Overall, this study describes a novel combination of PhotoVoice and digital stories to create a community-tailored prenatal health curriculum aiming to increase prenatal health knowledge, improve awareness of pregnancy danger signs, and address community-specific barriers to seeking care during pregnancy in the Parinari District.

## Methodology

**Fig 1:**
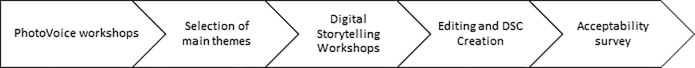
Digital story curriculum creation process.

This study had a sequential exploratory mixed-method study design that used qualitative methods for the process of digital story curriculum creation and quantitative methods for evaluation of digital story acceptability (see Fig 1 for a schematic of the study). The research article was written according to the guidelines for writing manuscripts about CBPR [24] and the STROBE guidelines for cross-sectional studies [25].

### Study setting

Parinari is a district in the Loreto region that has 30 communities [26] with approximately 7264 people [27]. The communities are very dispersed and only accessible by river. The predominant population belongs to the Kukama-Kukamiria ethnic group. Their main commercial activities are fishing, horticulture and the extraction of fine woods. They also sell various products, such as rice, cassava, bananas, corn and beans [28]. Inhabitants of this district do not have access to running water, electricity or sanitation and between 62.5% and 74.0% of the population lives in poverty [27].

Our study took place in the 13 communities of Parinari where the Mama River program operated. These communities were selected by Mama River as they had active community health workers (CHW) and functioning cell phone service. The CHW are community members who can read and write with basic primary or secondary education. They are elected by their communities and have prior training from regional health authorities and a church organization. Briefly, Mama River selected CHW who were able to use smartphones in these communities and trained them on the use of smartphones to collect information of new pregnancies, deliveries, alarms signs or deaths of pregnant women or newborns. This information was later sent by CHWs to mobile and local health care providers. The training also included how to deliver verbal MCH educational messages to pregnant women but did not use cell-phones for the delivery of MCH educational messages or videos.

### Photovoice

The primary investigator (PI) ran three community-based PhotoVoice workshops with 17 Mama River CHWs and 10 women who were invited by the CHWs. The CHWs had invited women with prior pregnancy experience, approximately between age 18–55, and ten women elected to attend. One government health worker who worked as a nurse at the local community health center was also present at one of the workshops.

The workshops followed the PhotoVoice guidelines [29], and began with a session on the concept of PhotoVoice and practicing basic photo taking using mobile phones and donated digital cameras. During the workshop, the process behind PhotoVoice, and examples from other projects were shared. Then, participants were taught basic photography techniques, such as how to focus the camera, how to best set the lighting, and how to frame a shot. Next, the groups discussed the ethics of photography, when it is acceptable to take a photo of someone else, and how to ask for consent. Finally, all participants were instructed to take photos that represented answers to the following three questions:

1. *What does a healthy pregnancy mean to you*?
2. *What are the positive aspects of your community that help women have healthy pregnancies*?
3. *What are the barriers to having a healthy pregnancy in your community*?

Participants had two weeks to take photos, and select the ones that they felt were the most meaningful. These photos were then printed back in Lima by members of the study team, and brought back by the PI to the communities.

**Fig 2:**
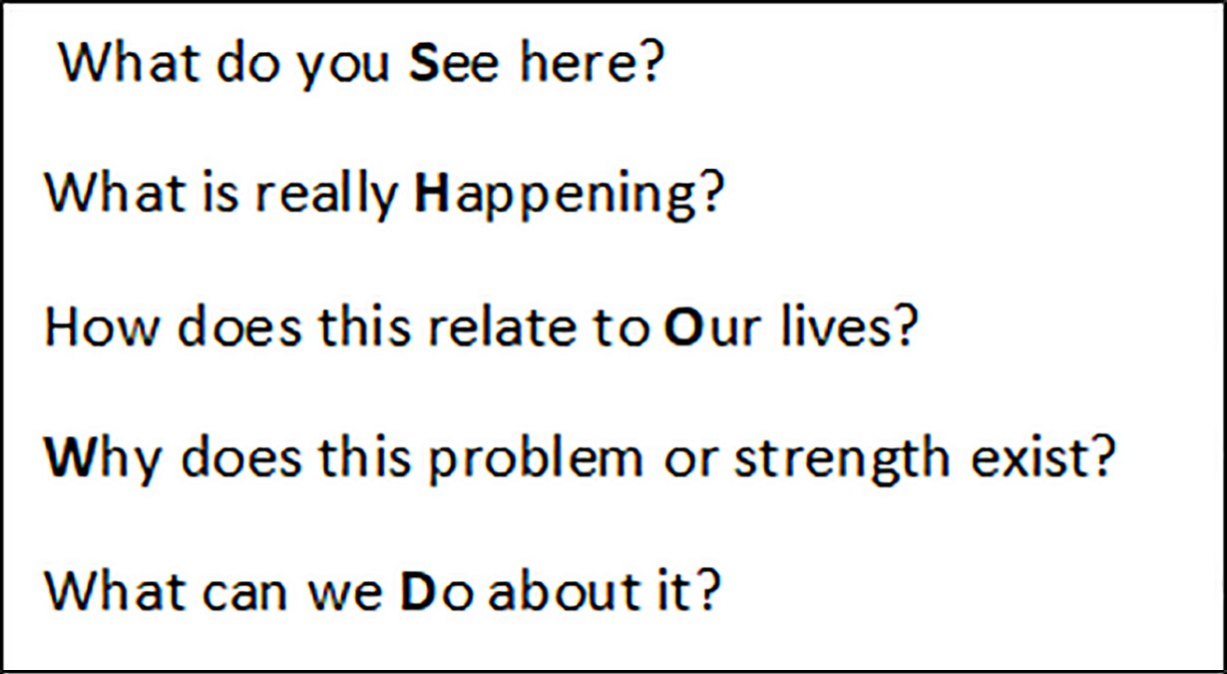
SHOWeD method [30].

A second set of workshops was then conducted with the same participants, in which they discussed the full printed set of photos. Participants first discussed all possible messages within each photo, and then the photo-taker shared his/her intended message. Group discussions were based around SHOWeD method [29], a standardized set of questions used in PhotoVoice workshops (Fig 2). These questions were translated into Spanish and verified by three bilingual team members.

Participants wrote messages describing their reasoning for taking the photos, and shared them during group discussions. The resulting photos and messages enabled the selection of community-specific themes related to MCH.

### Digital storytelling

Using the list of themes developed above, the PI then led community-based digital storytelling activities which were designed based on published guides [31,32]. The same groups of participants engaged in storytelling exercises, using the themes to brainstorm experiences during their own, their partner’s or child’s pregnancy. Participants were encouraged to think of personal stories and recorded themselves on mobile devices narrating their stories. Then participants did a storyboarding activity, in which they broke down the components of their story, and determined what types of photos would fit with each component of the story. Participants had 3–7 more days in which to take further pictures to tell their story. Written informed consent was obtained for all persons photographed or recorded using a consent form approved by the IRB of the Universidad Peruana Cayetano Heredia. The consent included permission to use all media collected for print, publication, online dissemination, and for inclusion in Mama River program materials. Participants also suggested other community members who had personal stories relating to these themes. In these cases, the participant then accompanied the PI to approach these community members, who were then formally consented for participation and audio recording.

### Digital story curriculum creation

From these recorded stories, the research team selected the ones that met the quality criteria of recording quality and intelligibility of speech, and then further selected based on relevance to the themes identified during Photovoice workshops and the clarity of the storyline. The team worked with a professional filmmaker and a professional midwife to develop these stories into a final digital story curriculum (DSC), where each story was combined with question-based teaching prompts. The research team and community leaders chose the name “Nuestras Historias” or “Our Stories” for the DSC, as this best represented what the content meant to all those who participated. The curriculum of seven digital stories was loaded onto solar-charged tablets. The PI then trained 27 CHW on how to use the tablet devices, show the digital stories, and lead discussions on the lessons from the stories with pregnant women and their partners in both individual and small group sessions of 2–5 participants. One additional male CHW received the same training from a CHW leader because he couldn’t be present during the sessions with the PI. Finally, a total of 28 CHW (79% female and 21% male) were trained in DSC use.

### DSC acceptability

Community members’ reactions to the digital stories were then measured using a survey instrument. The survey contained questions derived from other studies evaluating video curricula [16,33] and a short version of a validated Transportation Scale, which evaluates the level of engagement with narrative stories [34,35]. The survey was assessed for content validity by three experts in the fields of public health research and women’s health. After informed consent was obtained, the research team used a small solar-charged tablet to show the digital stories to male and female survey participants within the Parinari area. These participants were selected purposively based on falling within the appropriate age range for the curriculum’s intended eventual audience of pregnant mothers and expecting fathers. This part of the study served to assess the understandability of the digital stories and the relevance, and emotional impact of the stories on community members who watched them.

### Ethical considerations

The design phase protocol and instruments were approved by the Institutional Review Board (IRB) of Universidad Peruana Cayetano Heredia (SIDISI 65203), and waived by the University of Washington IRB. The protocol of the acceptability survey instrument was approved by both review boards (UPCH SIDISI 65203 and UW Human Subjects 51617).

## Results

### PhotoVoice

**Fig 3:**
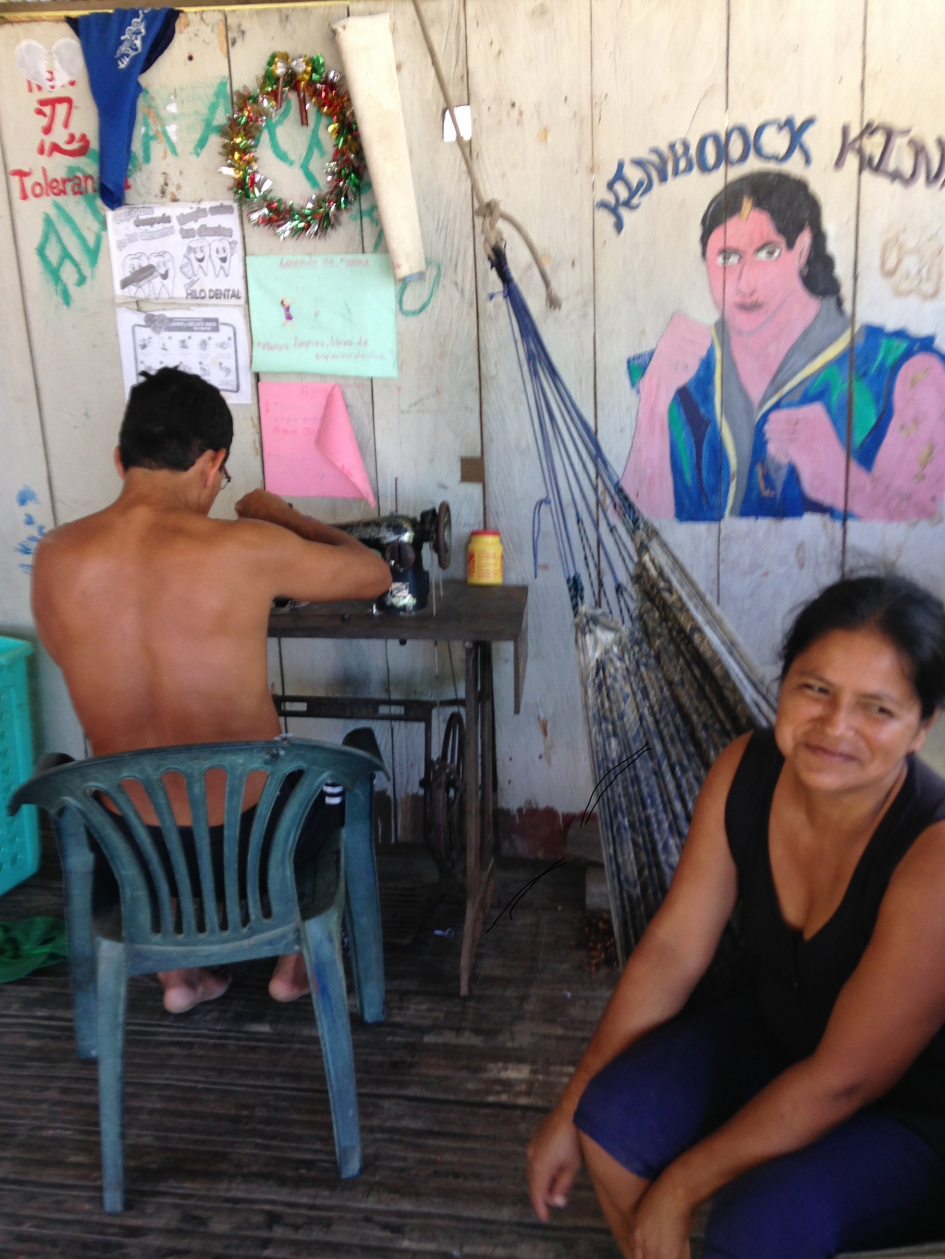
“This photo shows a husband helping his pregnant wife with sewing. A husband’s support is so important during pregnancy, but many women do not have it”.

**Fig 4:**
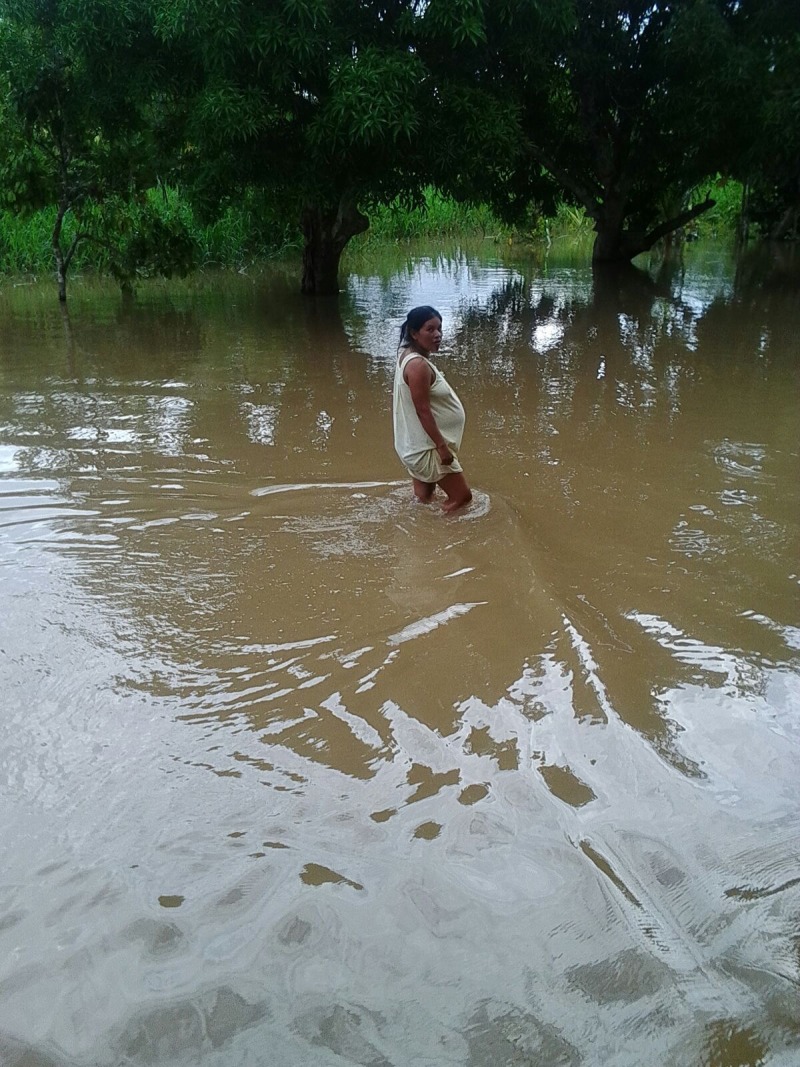
“A pregnant woman is walking in the water- she is walking from one house to another, returning from the home of the traditional birth attendant during the rainy season”.

**Fig 5:**
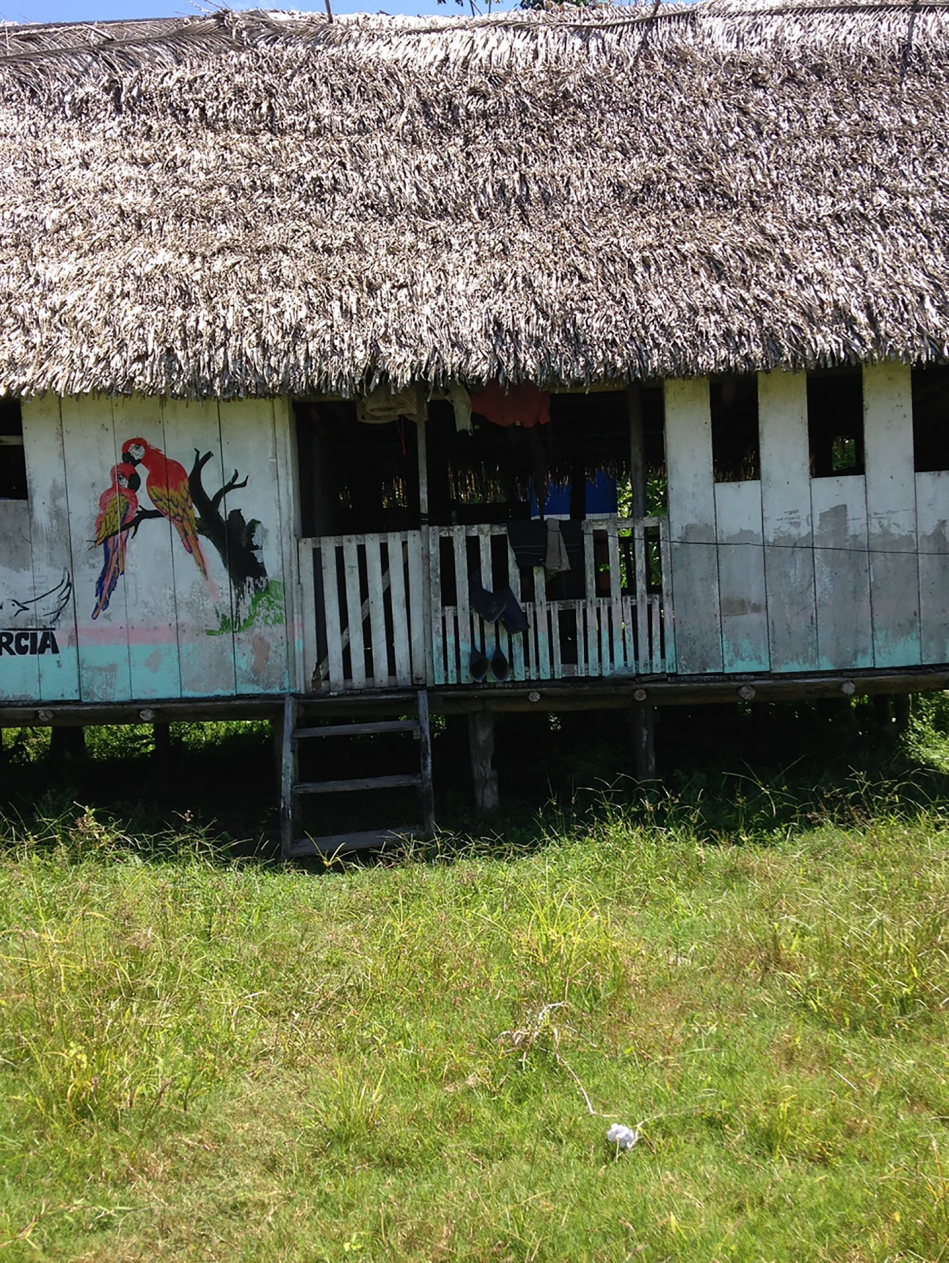
“This house can be compared to a mother, a mother who is alone, going through pregnancy alone, sad and abandoned with no one helping her”.

**Fig 6:**
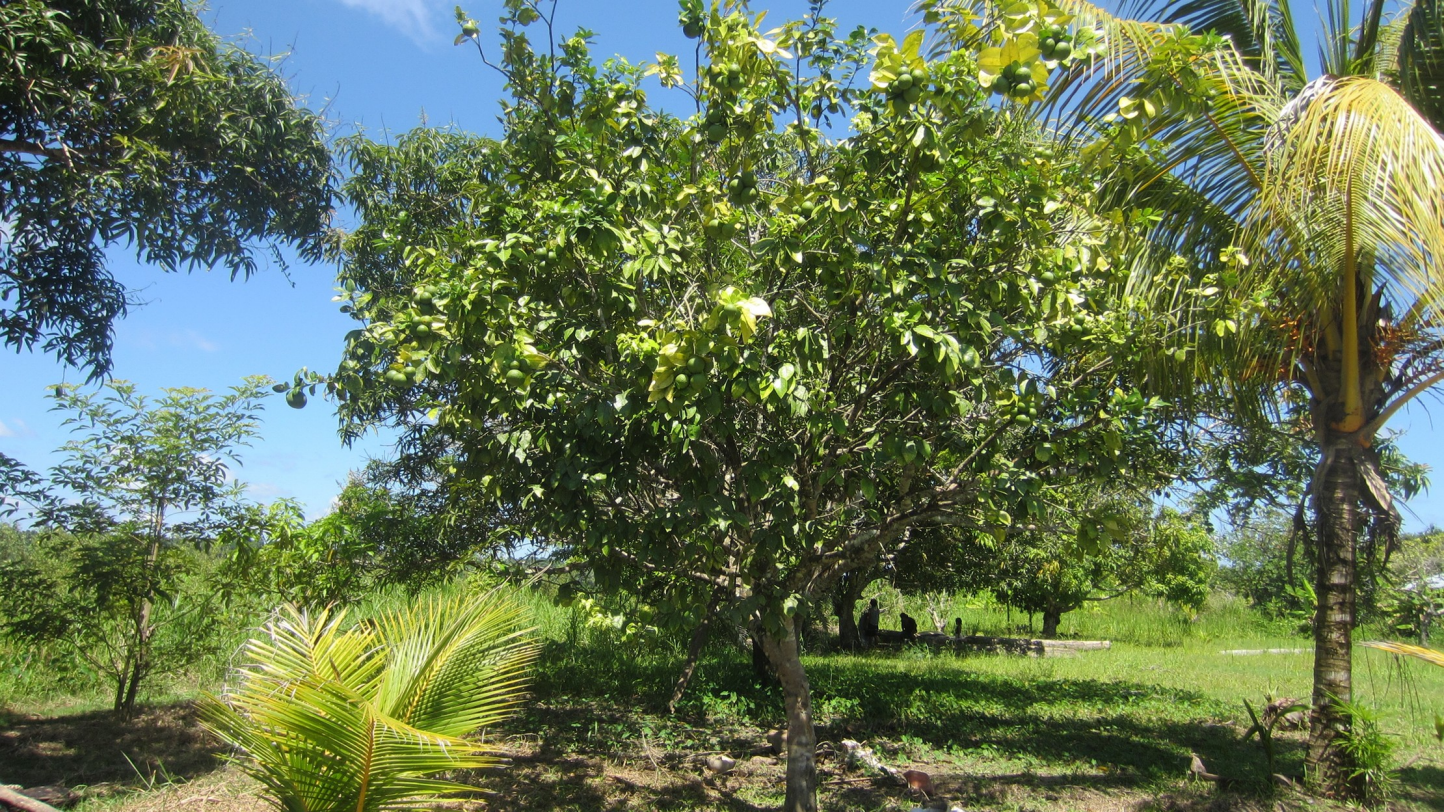
“Orange- this is a little tree that is well cared for, and it can be compared to a mother who has done everything she should- her prenatal care appointments, to have a good pregnancy, a well-developed baby, just like this tree with its healthy oranges. There are no risks, and just healthy births”.

Three initial workshops were conducted involving a total of 17 CHW and 10 mothers from the 13 Mama River communities, along with one local government nurse. Participants were all trained in PhotoVoice and took photos on a variety of themes related to maternal health (Figs 3–6).

**Fig 7:**
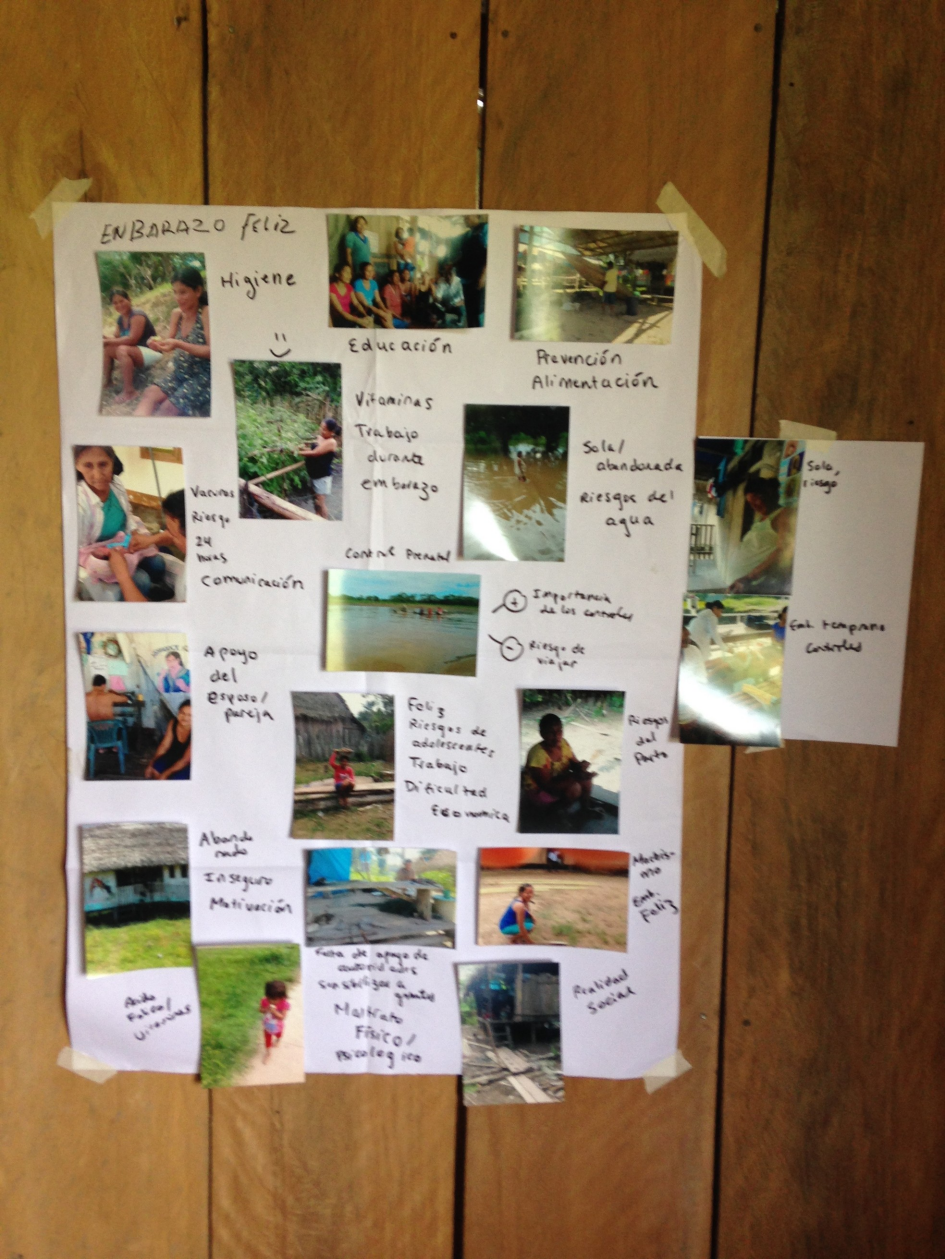
Using participants’ photos to create a list of pertinent themes and issues during pregnancy, taken during the second phase of PhotoVoice workshops in Santa Rosa. Written on this poster (from top left to right bottom): -Happy pregnancy, hygiene, education, prevention, nutrition and vitamins, labor during pregnancy, abandonment during pregnancy, risks of the water/travel, vaccines, risks at 24 hours, importance of prenatal health care, going early to get prenatal care, being alone, support from the partner, risks of adolescent pregnancy, financial struggles, risks during birth, insecurity, lack of vitamins, psychologic and physical abuse, social realities.

A second set of three workshops was then conducted with the same participants, in which the photos and messages were discussed. Participants guessed the messages of others’ photos, and shared their own messages. The group discussion of the photos and messages led to the creation of a list of themes (see Fig 7 for an example of the discussion of themes in one PhotoVoice group), which were then pared down to a final list. The themes from the three separate workshops had significant overlap, and were compiled and used to determine the focuses for the education curriculum. The major themes identified as barriers to healthy pregnancy that were consistent across the three groups were as follows:

- Complications and illnesses during pregnancy
- Lack of partner support
- Domestic abuse
- Adolescent pregnancy
- Complications during delivery
- Accessing vaccinations and prenatal care
- Division of labor during pregnancy

### Digital story curriculum creation

**Fig 8:**
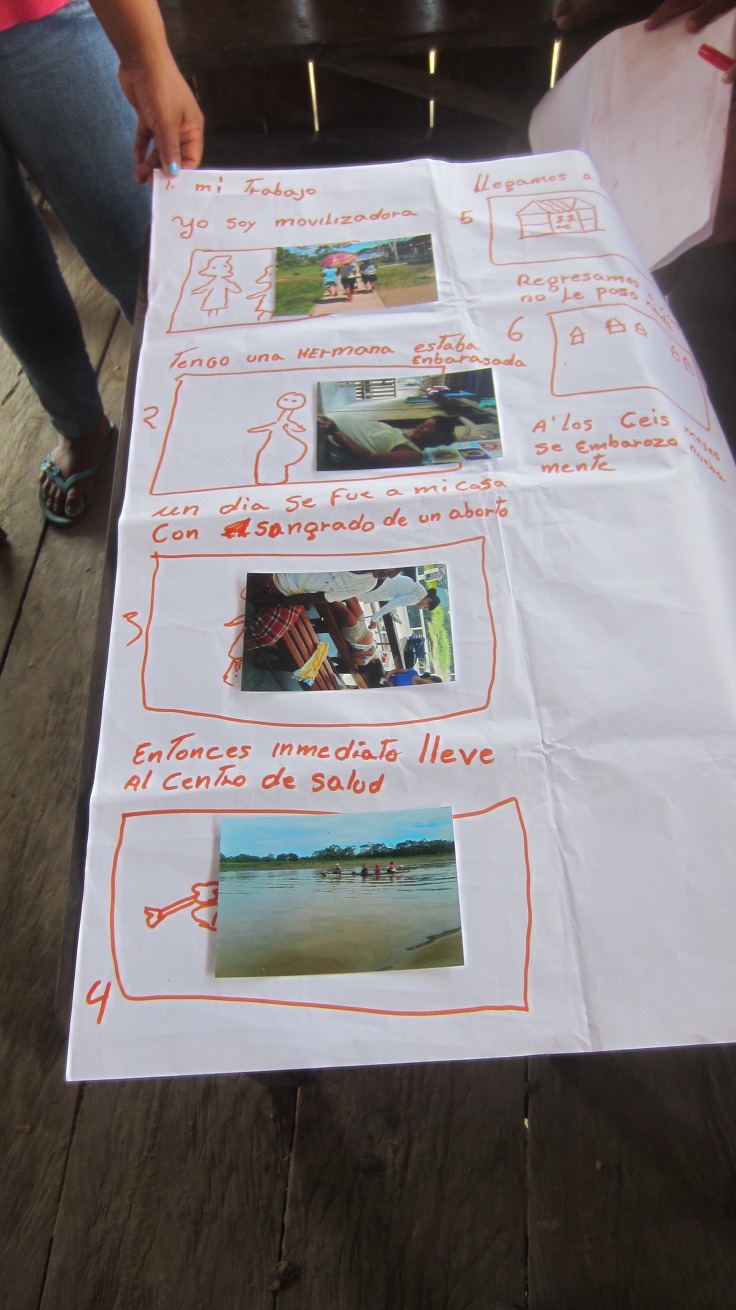
A participant storyboards, selecting photos that best illustrate her story.

**Fig 9:**
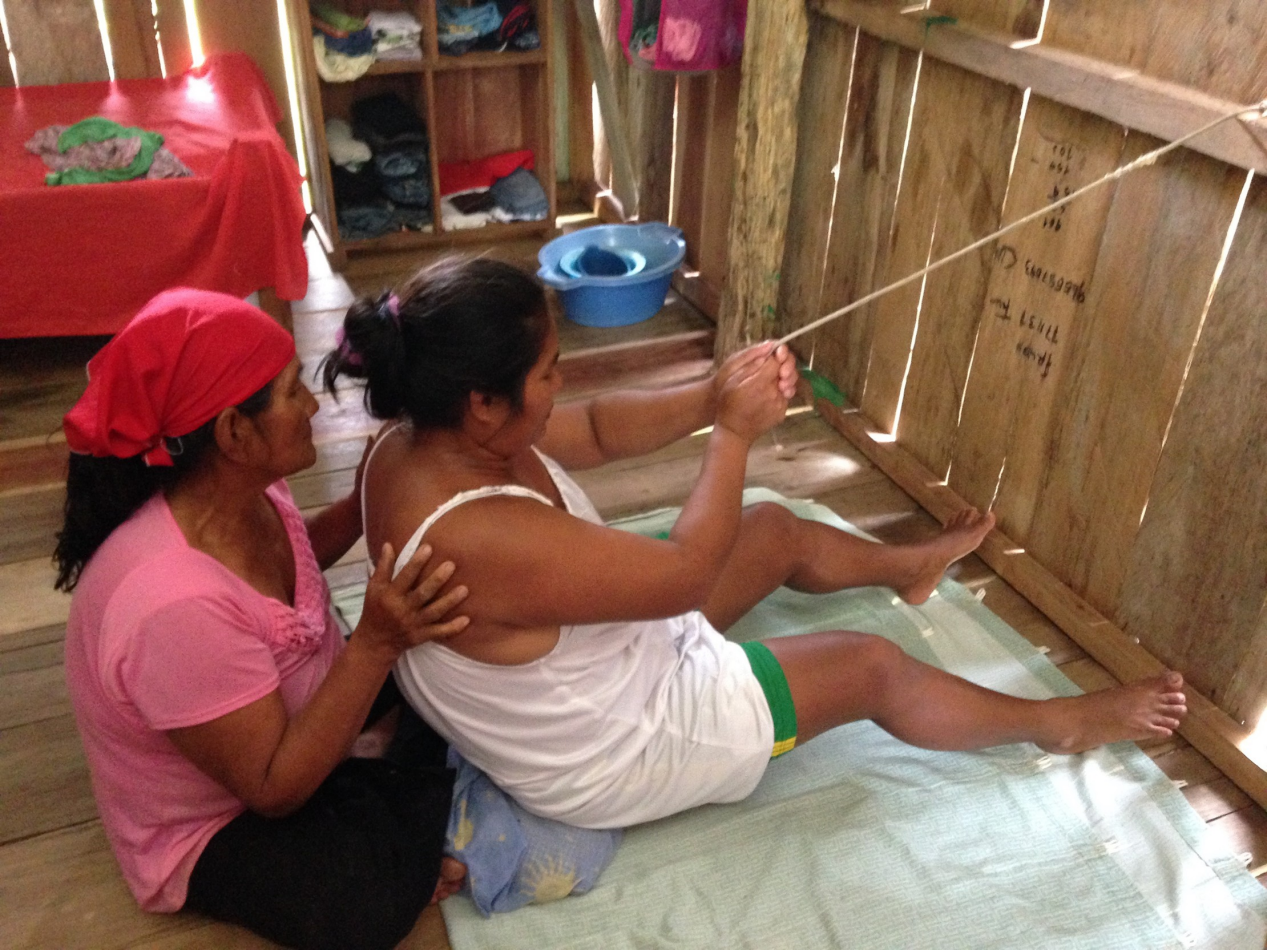
Participant acting out scene of a home delivery with her mother for her digital story.

The participants then brainstormed personal stories, or stories within their families, related to these themes. For example, one story describes the struggle to get money for gas for the canoe motor needed to get to a health center. Another includes the process of squatting and holding a rope when attempting to deliver a baby at home, which is acted out in a photo by a community member. Participants recorded their stories, and then created storyboards incorporating photographs that fit well with their story arcs (Fig 8). Participants also took additional photos for their stories, ranging from metaphoric representations of story components and some photos of community members acting out scenes from the story (Fig 9). Some participants also knew of other community members that had relevant experiences, and those people were also approached for possible recordings.

In total, 31 stories were recorded and over 500 photographs were taken during the storytelling and storyboarding process. The PI listened to all the recordings and edited them down to the typical digital story length of 3–5 minutes. After selecting 15 which met the quality control criteria, the full research team again listened to select the set of stories that best followed a single narrative storyline and fit best with the initially identified themes. After this second process, seven stories were selected that adequately covered the above listed themes. The team then worked with a videographer to take additional photos and combine the photos with audio to create the set of digital stories. An educational component was added at the end of each story, with teaching points based on the section of the Peruvian government’s guide for community health workers [36] that fit best with the story’s theme, and known misconceptions that had come up during the workshops and prior Mama River fieldwork. The seven final digital stories are summarized in Table 1, and come together as the curriculum called Nuestras Historias. Five of them received pseudonyms to preserve the privacy of the participants, but two CHWs requested the PI not to change their names.

**Table 1:** List of videos of the digital story curriculum.

Name	Digital Story Synopsis	Teaching Points
**Pepe** (Community health worker) The value of prenatal care	Pepe’s wife had three healthy pregnancies with no prenatal care, but in the fourth had severe abdominal pain at home in the last month of her pregnancy. The baby stopped moving. Pepe struggled to get his wife to the health center, and the infant unfortunately passed away before delivery. He asks others to seek out care sooner to avoid such outcomes.	• What alarm signs did Pepe’s wife have? • What are the other alarm signs during pregnancy? • Why is prenatal care important?
**Celia** (Community health worker) The risks with home deliveries	In Celia’s second pregnancy, she initially did not realize she was pregnant, and then was delayed in getting prenatal care. She decided to deliver the baby at home, but had a prolonged course of labor. In severe pain, she begged her mom to take her to the health center, but it was too late, and she lost consciousness. Thankfully, her parents were able to deliver the baby at home safely. After this scary event, she decided she would have all other children at a health center, and encourages all other women to do the same.	• When should you go for your first prenatal appointment? • What are the alarm signs during delivery? • What are the benefits of giving birth in a health center? • How can you confirm that you are pregnant? • Did Celia have a birth plan? What is your birth plan?
**Mariela** (named changed) Domestic violence during pregnancy	Mariela separated from her partner, but one night he came back, intoxicated, and violently raped her. She became pregnant from this rape, and 8 months into her pregnancy, he appeared and attacked her again. She feared for the life of herself and the fetus, but was too afraid to report the incident and worried about the stigma. She and the baby are safe and well now, but she motivates others to speak up and use their voices to report men and end other such incidents.	• How can abuse affect a pregnancy? • How can we confront the issues of domestic violence and abuse in our community?
**Victor** (name changed) A reflection on teen pregnancy	Victor describes his first relationship, when he and a girl from school were both thirteen. When they had sex for the first time, she got pregnant. She became very sick at the end of her pregnancy, and her family had to take her to the hospital. It was a long boat journey, and she and the baby died on the way. Her family was furious at Victor, and he did not know what to do. He ended up leaving his hometown, and has never been back. He encourages teens to learn more about reproductive health, so that things will be different today.	• Why is pregnancy during adolescence riskier? • How can we avoid teen pregnancies in our community?
**Carla** (name changed) Recognizing danger signs in pregnancy	Carla’s daughter was sixteen and in her eighth month of pregnancy. She was complaining of headaches the night that her grandfather passed away. At the wake, her headaches got worse. Everyone felt it was related to her grief from the death, but then, she began seizing. The family took her to two local different healers, who agreed that it was a “choque de aire”- a shock of air, likely from grief. But then one family member realized her headaches and seizures were from hypertension. They mobilized, and were able to get her to the health center and then hospital on time. The baby and mother survived, though the baby has some developmental delays.	• What alarm sign did Carla’s daughter have? • What can this alarm sign, and hypertension, lead to? • What should you do if you have a headache during pregnancy?
**Rosa** (name changed) Lack of partner support and hard labor during pregnancy	Rosa was in her seventh month of pregnancy when she was working out in the fields. She came home carrying a heavy load of bananas, and slipped and fell down hard. Soon after, she noticed that her abdomen was becoming harder, she had foul smelling discharge, and the baby had stopped moving. She wanted to go to the clinic, but it was the dry season and was hard to reach the water with no help. A midwife helped take care of her, but she delivered a still-born. She reminds others that a woman’s role is hard and there is sometimes no one to help out.	• What activities are safe during pregnancy, and what should be avoided? • What should you do if you feel the baby is no longer moving? • How can your partner support you during pregnancy? • How can we improve the problems we have with transportation?
**Juana** (name changed) The importance of immunizations	Juana and her neighbor were pregnant at the same time. An immunization campaign was passing through the village, and Juana got vaccinated, but her neighbor was too busy working in the fields. Her neighbor delivered first, but her baby contracted tetanus. Juana had gone to help take care of the baby, and was very fearful that her own baby would be sick as well. Thankfully, she delivered a healthy baby, and then rowed several hours to get to her first newborn care appointment. She reminds others of the importance of vaccination.	• What is tetanus? • What does the tetanus vaccine do? • What vaccines do babies need, and when?

### Assessing acceptability of the DSC

The acceptability of the DSC was assessed between April 2016 and February 2017 during two fieldwork trips to the Parinari communities. In total, 60 females and 47 males were surveyed, with each participant viewing between one and two digital stories. Each digital story was viewed by at least 10 participants. The demographics for the participants are shown below in Table 2. The mean age for male participants was 40.1, and the mean age for females was 32.8. The majority had some secondary schooling, had health insurance provided by the government, and had no specific role in the community.

**Table 2:** Demographics of survey participants.

	Male N = 47 (43.9%) n (%)	Female N = 60 (56.1%) n (%)
**Mean age (SD)**	40.2 (10.7)	32.8 (10.1)
Education Level*		
Primary School	20 (43.3)	28 (47.5)
Secondary School	24 (52.2)	30 (50.9)
University	2 (4.4)	1 (1.7)
**Mean number of years in school (SD)**	8.3 (2.9)	7.2 (2.6)
**With health Insurance**	41 (89.1)	58 (98.3)
**Role in Community**		
Common Citizen	21 (44.7)	47 (78.3)
Community Health Worker	2 (4.3)	1 (1.7)
Authority	16 (34.1)	4 (6.7)
Other	8 (17.0)	8 (13.3)
**Family Member with Role in Community**	17 (36.2)	20 (33.3)

*Only 46/47 male and 59/60 female participants responded to this question

All participants were shown the digital stories, and then asked to answer a series of survey questions. The full results of their reactions to the digital stories are shown in Table 3. Overall, the vast majority of participants felt that the digital stories were novel, relatable, educational, and shareable. All men and women (100%) rated the digital stories as “good” or “excellent,” and the large majority felt the videos were useful, understandable, and relevant as well.

**Table 3:** Survey reactions to digital stories.

	Male	Female
	(N = 47, 43.9%)	(N = 60, 56.1%)
**Reactions to Digital Stories**	**n (%)**	**n (%)**
**Novel:** I have never seen a video like this	42 (89.4)	50 (83.3)
**Relatable:** I could relate well to the story in the video	42 (89.4)	55 (93.2)*
**Educational**: I learned something new from the video	43 (91.5)	56 (93.3)
**Shareable:** I would share this video with others	47 (100.0)	60 (100.0)
**Overall, I felt the video was**		
Excellent	26 (55.3)	22 (36.7)
Good	19 (40.4)	35 (58.3)
Fine	2 (4.7)	0 (0.0)
Bad	0 (0.0)	3 (5.0)
**In terms of usefulness, the video was**		
Very Useful	31 (66.0)	31 (51.7)
Useful	13 (27.7)	27 (45.0)
Somewhat Useful	2 (4.3)	2 (3.3)
Not Useful	1 (2.1)	0 (0.0)
**I could understand the video**		
Completely	17 (36.2)	27 (45.0)
Somewhat	15 (31.9)	13 (21.7)
A little bit	15 (31.9)	20 (33.3)
Not at all	0 (0.0)	0 (0.0)
**In terms of relevance to our community, the video was**		
Very relevant	33 (70.2)	39 (65.0)
Relevant	14 (29.8)	19 (31.7)
Somewhat relevant	0 (0.0)	1 (1.7)
Not at all relevant	0 (0.0)	1 (1.7)

*Only 59 of the 60 female participants responded to this question

**Fig 10:**
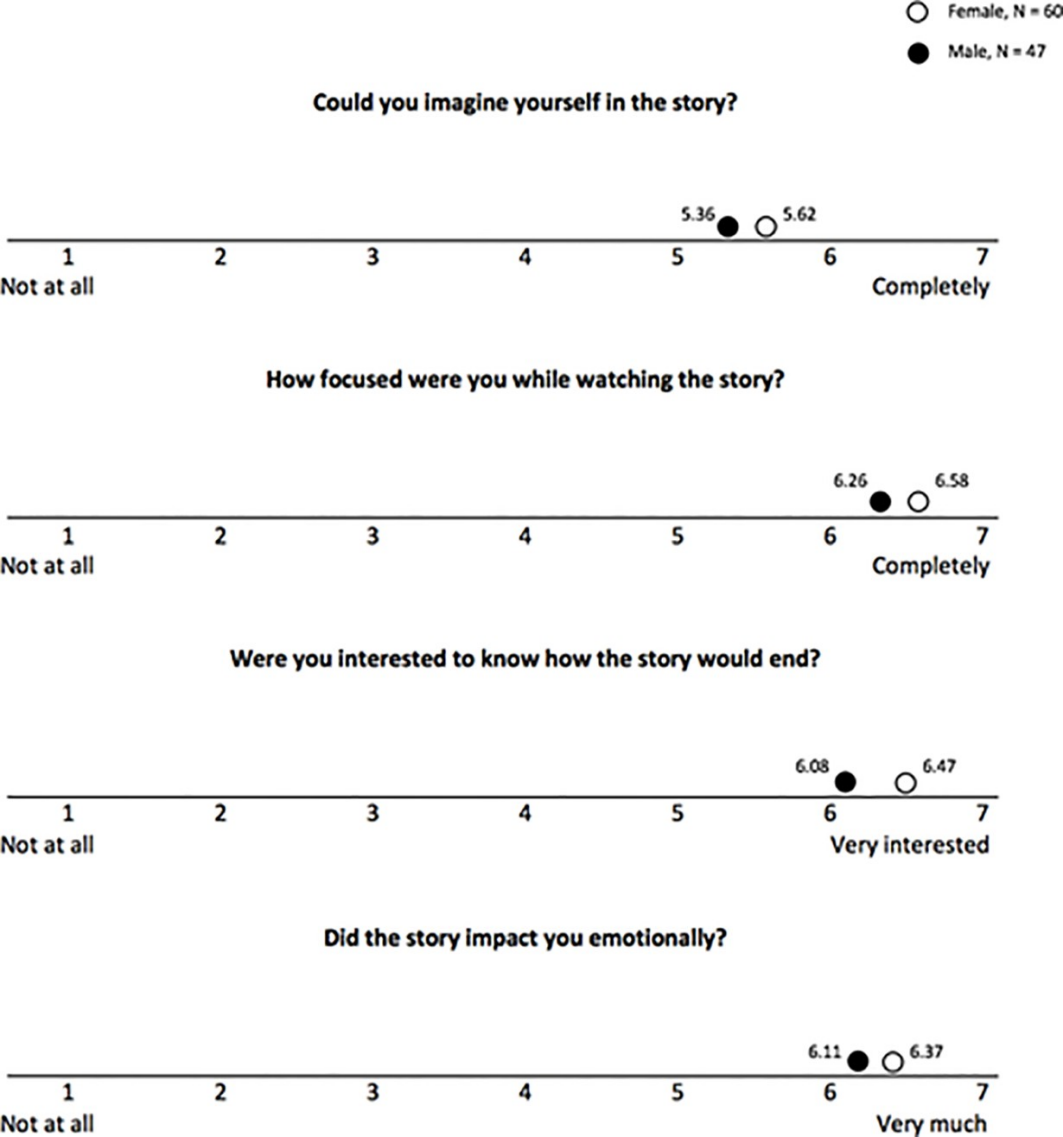
Results of transportation scale-short form for digital stories, mean scores.

Participants also completed a transportation scale after viewing the digital story, which measured their engagement and focus with the narrative. One element of the five-point scale (“How well could you imagine the narrator of the story?”) was removed during the pilot study, as participants universally had difficulty understanding the meaning of the question prompt. Fig 10 shows male and female participants’ responses to remaining four points on the transportation scale (Fig 10). Overall, both male and female participants showed a high level of transportation into the narrative.

## Discussion

The results from this project demonstrate the potential of using CBPR in low-resource settings with low-literacy population to develop novel behavior change interventions, specifically the use of digital stories as a health promotion tool for MCH. PhotoVoice was key to the development of this intervention, empowering community members to share their unique views on both positive aspects of pregnancy and the challenges encountered. This methodology allowed participants who were less vocal and those who could not read and write, who otherwise may not have been able to express their opinions, to fully participate. The PhotoVoice process enabled investigators to better understand the MCH issues specific to the region, and led to the selection of DSC topics that somewhat differ from the traditional components of prenatal health education curriculums.

The World Health Organization’s latest guidelines for MCH suggest that lay health workers cover prenatal health promotion topics including the following: appropriate care seeking, prenatal care in pregnancy, companionship during labor, birth preparedness, skilled childbirth, adequate nutrition, reproductive health and family planning, immunizations, newborn care and care of low birth weight infants [37]. Studies on prenatal education interventions in rural settings appear to focus most on danger signs and birth preparedness [38–41]. The DSC did cover many of these themes, as they were raised as key issues during the workshops, but the PhotoVoice process also allowed the DSC to include other locally significant themes. For example, in Loreto, adolescent pregnancy is very common, and 30.6% of adolescents between 15 and 19 years in the region have been pregnant at least once [3]. Community members identified this as a pertinent issue, and one digital story is therefore dedicated to the issues that pregnant adolescents and adolescent partners can face. Domestic violence also revealed itself during the workshops as a significant issue and because of this, one digital story is a testimony about domestic violence and addresses abuse and mistreatment in pregnancy.

Even within the digital stories addressing more traditional topics, such as the importance of prenatal care or safe delivery, the narrations include many details about the realities of local river transportation, the involvement and practices of traditional healers, and local customs during deliveries. These local issues are critical to discuss and were expressed due to the processes of digital storytelling, of recording and photographing the true personal stories of community members in rural Loreto, truly making the curriculum fit its name of Nuestras Historias- Our Stories. The combination of PhotoVoice and digital storytelling demonstrate that communities have unique MCH needs that may be best revealed and addressed through CBPR and community-created interventions. The research team is currently addressing how to include topics that are essential in the WHO guidelines but did not come up through the PhotoVoice process, specifically around newborn care. The team has already conducted additional PhotoVoice and storytelling sessions around this topic, and has also been filming videos on newborn care narrated by a local well-known midwife.

PhotoVoice and digital storytelling have been combined in the past for needs assessments and advocacy work [23,42,43], but this is one of the first studies that uses the combination in order to create a public health intervention, and the only existing study that documents the creation of a digital story-based maternal health intervention and assesses its acceptability. The main strength of the methodology is that the entire curriculum was designed using participatory community-based methods, leaving no question as to whether the curriculum would apply well to the community. This was confirmed by the highly positive feedback from the acceptability survey, in which an overwhelmingly large majority of both male and female participants reported that the digital stories were novel, relatable, educational, and shareable. The inclusion of males in the piloting was highly important, as the influence of males on decisions made during pregnancy is well-known, particularly in more rural and more patriarchal societies [44]. At present, there are few rigorous studies evaluating the impact of targeting men in MCH programs [44–46], but this initial pilot shows the feasibility of engaging them in the DSC.

While the results are very promising, the CBPR methods used also have limitations that the researchers were careful to acknowledge. In an ideal setting, PhotoVoice enables participants to share personal, genuine views, but too much supervision during the use of the cameras can limit this aspect [17,19]. During the initial workshops, the facilitator tried to avoid giving examples of photos specific to the prompts in order to avoid influencing the participants’ photo choices. Similarly, facilitators were not present during the process of the photo-taking. However, even with these precautions, there was some priming and influence between participants, and some of the more experienced CHWs ended up instructing the lower level health workers and community women on what photos to take. In terms of the ethics of photographing others, participants went through a basic consent training, and were required to ask permission before taking any photo of any person. The process of asking for consent is necessary but can take away from the organic nature of photography. Every identifiable person that appeared in a photograph had to sign an informed consent allowing use of their images in videos and presentations. After the photographs were selected and discussed and the main topics were identified, people were encouraged to share their experiences and thoughts. Once again, the more vocal CHWs often shared first, and may have guided others’ opinions, but every effort was made to have all participants share their views.

A major limitation during digital story development was that it was not feasible to involve storytellers in each step of the process. Ideally, during the creation of a digital story, the storyteller is able to select photos, edit the audio, and create the story themselves on a computer [42]. This was not possible due to limitations in technical resources and time, specifically the ability to edit audio and photos in the communities when participants had no computer literacy and there was no consistent electricity to charge computers. Instead, participants were involved in voice-recording and initial storyboarding, but the photos and audio were then brought into the project office in Lima to be edited and spliced. This also meant that though the themes were chosen by the community, the final stories were selected by the research team alone, which takes away from the participatory nature of the project. For stories that did not have enough corresponding photos from the workshops, additional photos were taken by the research team with direction from a filmmaker. The PI attempted to get approval from the storytellers for all additional photos that were added. However, one storyteller did not want to be involved beyond telling the story, so all photos, editing and splicing were done by the team alone.

It is important to mention that the survey could be affected by desirability bias, because the same team that was involved in the workshops also performed the survey. Though there was no overlap between workshop and survey participants, the survey participants likely recognized and knew the research team had been involved in the digital story creation. Ideally, another not-related team should have done the acceptability survey to avoid this kind of bias, but it couldn’t be possible due to resource constraints.

Finally, though the survey results were overall positive, and the majority of participants reported they could understand the stories, close to 1/3 reported they only understood “a little bit”. Investigators noted that participants were generally able to summarize the stories well, and it is possible some reporting of only “some” understanding was a way to be humble that is culturally common. Some videos also had narrators that spoke very quickly, which could have limited some understanding. Also, there were many distractions and noise while watching the videos including children running in and out and rainy weather when the interviews were done outside. These were the realities of the community setting, but ideally should be better accounted for in future research.

Although there were limitations, these digital stories are the first attempt to create customized, culturally-specific educational material in maternal and child health with the Kukama-Kukamiria people of Parinari. The evaluation of the curriculum’s behavior-change potential will be addressed in further publications. The DSC is already being used by some CHWS who participated in the pilot, and they have already been incorporated into the Mama River mobile application, so that it can be used by CHWs during home visits to educate and motivate pregnant women and their families on making healthy pregnancy decisions.

## Conclusions

Nuestras Historias is an innovative educational strategy that addresses community-specific problems around maternal and child health through narrative persuasion using local voices and photography. The Nuestras Historias curriculum is highly engaging and highly accepted by both men and women in the target population, and the package of seven videos will be further evaluated through a cluster randomized trial. This study proves the feasibility of conducting participatory research using Photovoice and digital storytelling in rural indigenous communities in the Peruvian Amazon with populations with lower literacy levels and without access to basic services such as electricity. The combination of PhotoVoice and digital storytelling can serve as a model to inspire the development of other behavioral change interventions in a community-tailored manner, both for MCH and for broader public health issues.

## References

[1] Instituto Nacional de Estadística e Informática. Encuesta Demográfica y de Salud Familiar- ENDES 2013. INEI; 2013.

[2] Warren J, Lambert, Edelman, Fu, Anderson J. Global neonatal and perinatal mortality: a review and case study for the Loreto Province of Peru. Res Rep Neonatol. 2012 10;103.

[3] Instituto Nacional de Estadística e Informática. Encuesta Demográfica y de Salud Familiar- ENDES 2016. INEI; 2016.

[4] Wingfield T, Ramirez R, Williamson J. Health, Healthcare Access, and Use of Traditional Versus Modern Medicine in Remote Peruvian Amazon Communities: A Descriptive Study of Knowledge, Attitudes, and Practices. Am J Trop Med Hyg. 2015 4 1;92(4):857–64. 10.4269/ajtmh.14-0536 25688165 PMC4385786

[5] NU. CEPAL. Observatorio de Igualdad de género de América Latina y el Caribe. Mujeres indígenas en América Latina: dinámicas demográficas y sociales en el marco de los derechos humanos [Internet]. CEPAL; 2013. Available from: https://www.cepal.org/es/publicaciones/4100-mujeres-indigenas-america-latina-dinamicas-demograficas-sociales-marco-derechos

[6] Ministerio de Salud. Programa presupuestal Salud Materno Neonatal [Internet]. Ministerio de Salud; 2018. Available from: https://www.minsa.gob.pe/presupuestales2017/doc2018/pp/anexo/2/ANEXO2.pdf

[7] Limaye NP, Blas MM, Alva IE, Carcamo CP, García PJ. The Amazon Hope: A qualitative and quantitative assessment of a mobile clinic ship in the Peruvian Amazon. PloS one. 2018 6 21;13(6):e0196988 10.1371/journal.pone.0196988 29927934 PMC6013175

[8] Avellaneda RDSA Fontenele CV, Sena BF Diniz SG. Birth at the health center or at home: an analysis of birthing care among the Kukamas Kukamirias women of Peru. J Hum Growth Dev. 2013 12 30;23(3):322.

[9] Mechael PN. The Case for mHealth in Developing Countries. Innov Technol Gov Glob. 2009 1;4(1):103–18.

[10] Tamrat T, Kachnowski S. Special Delivery: An Analysis of mHealth in Maternal and Newborn Health Programs and Their Outcomes Around the World. Matern Child Health J. 2012 7;16(5):1092–101. 10.1007/s10995-011-0836-3 21688111

[11] Gurman TA, Rubin SE, Roess AA. Effectiveness of mHealth Behavior Change Communication Interventions in Developing Countries: A Systematic Review of the Literature. J Health Commun. 2012 5 2;17(sup1):82–104.

[12] Anderson ES, Wagstaff DA, Heckman TG, Winett RA, Roffman RA, Solomon LJ, et al Information-motivation-behavioral skills (IMB) model: Testing direct and mediated treatment effects on condom use among women in low-income housing. Ann Behav Med. 2006 2;31(1):70–9. 10.1207/s15324796abm3101_11 16472041

[13] Tuong W, Larsen ER, Armstrong AW. Videos to influence: a systematic review of effectiveness of video-based education in modifying health behaviors. J Behav Med. 2014 4;37(2):218–33. 10.1007/s10865-012-9480-7 23188480

[14] Desta BF, Mohammed H, Barry D, Frew AH, Hepburn K, Claypoole C. Use of Mobile Video Show for Community Behavior Change on Maternal and Newborn Health in Rural Ethiopia. J Midwifery Womens Health. 2014 1;59(s1):S65–72. 24588918 10.1111/jmwh.12111

[15] DeStephano CC, Flynn PM, Brost BC. Somali prenatal education video use in a United States obstetric clinic: A formative evaluation of acceptability. Patient Educ Couns. 2010 10;81(1):137–41. 10.1016/j.pec.2009.12.003 20071131

[16] Scheinmann R, Chiasson MA, Hartel D, Rosenberg TJ. Evaluating a Bilingual Video to Improve Infant Feeding Knowledge and Behavior Among Immigrant Latina Mothers. J Community Health. 2010 10;35(5):464–70. 10.1007/s10900-009-9202-4 20039195

[17] Bisung E, Elliott SJ, Abudho B, Karanja DM, Schuster-Wallace CJ. Using Photovoice as a Community Based Participatory Research Tool for Changing Water, Sanitation, and Hygiene Behaviours in Usoma, Kenya. BioMed Res Int. 2015;2015:1–10. 10.1155/2015/903025 PMC4561937 26380305

[18] Castleden H, Garvin T, First Nation H. Modifying Photovoice for community-based participatory Indigenous research. Soc Sci Med. 2008 3;66(6):1393–405. 10.1016/j.socscimed.2007.11.030 18191883

[19] Wang C, Burris MA. Photovoice: Concept, Methodology, and Use for Participatory Needs Assessment. Health Educ Behav. 1997 6;24(3):369–87. 10.1177/109019819702400309 9158980

[20] Ghosh U, Bose S, Bramhachari R, Mandal S. Expressing collective voices on children’s health: photovoice exploration with mothers of young children from the Indian Sundarbans. BMC Health Serv Res [Internet]. 2016 11 [cited 2018 Mar 15];16(S7). Available from: http://bmchealthservres.biomedcentral.com/articles/10.1186/s12913-016-1866-8 10.1186/s12913-016-1866-8 PMC5123342 28185586

[21] Musoke D, Ndejjo R, Ekirapa-Kiracho E, George AS. Supporting youth and community capacity through photovoice: Reflections on participatory research on maternal health in Wakiso district, Uganda. Glob Public Health. 2016 7 2;11(5–6):683–98. 10.1080/17441692.2016.1168864 27109246

[22] Lal S, Donnelly C, Shin J. Digital Storytelling: An Innovative Tool for Practice, Education, and Research. Occup Ther Health Care. 2015 1 2;29(1):54–62. 10.3109/07380577.2014.958888 25338054

[23] Cueva M, Kuhnley R, Revels L, Schoenberg NE, Dignan M. Digital storytelling: a tool for health promotion and cancer awareness in rural Alaskan communities. Int J Circumpolar Health. 2015 1;74(1):28781 10.3402/ijch.v74.28781 26343881 PMC4561227

[24] Bordeaux BC, Wiley C, Tandon SD, Horowitz CR, Brown PB, Bass EB. Guidelines for Writing Manuscripts About Community-Based Participatory Research for Peer-Reviewed Journals. Prog Community Health Partnersh Res Educ Action. 2007;1(3):281–8. 10.1353/cpr.2007.0018 PMC4304664 20208291

[25] von Elm E, Altman DG, Egger M, Pocock SJ, Gøtzsche PC, Vandenbroucke JP, et al The Strengthening the Reporting of Observational Studies in Epidemiology (STROBE) statement: guidelines for reporting observational studies. J Clin Epidemiol. 2008 4;61(4):344–9. 10.1016/j.jclinepi.2007.11.008 18313558

[26] Gobierno Regional de Loreto. Mapa Político del Distrito Parinari [Internet]. [cited 2018 Apr 3]. Available from: http://geoportal.regionloreto.gob.pe/wp-content/uploads/2017/11/160302_MapaDistrito_PARINARI.pdf?656a5c

[27] Instituto Nacional de Estadística e Informática. Mapa de Pobreza Provincial y Distrital 2013 [Internet]. Lima: INEI; 2013 Sep. Available from: https://www.inei.gob.pe/media/MenuRecursivo/publicaciones_digitales/Est/Lib1261/Libro.pdf

[28] Ministerio de Transporte y Comunicaciones. Estudio de navegabilidad de los ríos Marañón y Amazonas: Tramo Saramiriza—Santa Rosa [Internet]. [cited 2018 Mar 4]. Available from: https://www.mtc.gob.pe/transportes/acuatico/documentos/estudios/Informaci%C3%B3n%20Socioecon%C3%B3mica.pdf

[29] Shama Mohammed, Sajun S. Photovoice Manual. Unpublished; 2014.

[30] Wallerstein N, Bernstein E. Empowerment Education: Freire’s Ideas Adapted to Health Education. Health Educ Q. 1988 12;15(4):379–94. 3230016 10.1177/109019818801500402

[31] Gubrium A. Digital Storytelling: An Emergent Method for Health Promotion Research and Practice. Health Promot Pract. 2009 4;10(2):186–91. 10.1177/1524839909332600 19372280

[32] Lunch N, Lunch C. Insights into participatory video: a handbook for the field. 1. ed Oxford: Insight; 2006. 125 p.

[33] Sureshkumar K, Murthy G, Natarajan S, Naveen C, Goenka S, Kuper H. Evaluation of the feasibility and acceptability of the “Care for Stroke” intervention in India, a smartphone-enabled, carer-supported, educational intervention for management of disability following stroke. BMJ Open. 2016 2;6(2):e009243 10.1136/bmjopen-2015-009243 26839011 PMC4746451

[34] Green MC, Brock TC. The role of transportation in the persuasiveness of public narratives. J Pers Soc Psychol. 2000 11;79(5):701–21. 11079236 10.1037//0022-3514.79.5.701

[35] Appel M, Gnambs T, Richter T, Green MC. The Transportation Scale–Short Form (TS–SF). Media Psychol. 2015 4 3;18(2):243–66.

[36] Ministerio de Salud. Documento Técnico: Preparando al Agente Comunitario de Salud para el cuidado integral de la salud y nutrición de las gestantes y de las niñas y niños menores de 5 años. (“El Manual del Agente Comunitario de Salud”) [Internet]. Lima, Perú; 2009. 174 p. Available from: http://bvs.minsa.gob.pe/local/minsa/1024_prom37.pdf

[37] World Health Organization, World Health Organization, Reproductive Health and Research. WHO recommendations: optimizing health worker roles to improve access to key maternal and newborn health interventions through task shifting. [Internet]. 2012 [cited 2018 Apr 30]. Available from: http://www.ncbi.nlm.nih.gov/books/NBK148518/23844452

[38] Shi Y, Wang D, Yuan Y, Jiang Y, Zeng Q, Chang C. The effect of prenatal education curriculum on mother’s prenatal examination utilization, delivery mode and recovery status: a cross-sectional survey in China. Environ Health Prev Med. 2015 11;20(6):397–403. 10.1007/s12199-015-0480-4 26201848 PMC4626461

[39] Ustunsoz A, Senel N, Pollock CA. Comparison of prenatal education delivered by nurses in Ankara (Turkey) and New Orleans (USA): Nursing role in prenatal education. J Clin Nurs. 2011 4;20(7–8):1133–40. 10.1111/j.1365-2702.2010.03262.x 20955477

[40] Mutanda JN, Waiswa P, Namutamba S. Community-made mobile videos as a mechanism for maternal, newborn and child health education in rural Uganda; a qualitative evaluation. Afr Health Sci. 2017 3 7;16(4):923. 10.4314/ahs.v16i4.6 PMC5398436 28479882

[41] August F, Pembe AB, Mpembeni R, Axemo P, Darj E. Effectiveness of the Home Based Life Saving Skills training by community health workers on knowledge of danger signs, birth preparedness, complication readiness and facility delivery, among women in Rural Tanzania. BMC Pregnancy Childbirth [Internet]. 2016 Dec [cited 2018 Apr 3];16(1). Available from: http://bmcpregnancychildbirth.biomedcentral.com/articles/10.1186/s12884-016-0916-x 10.1186/s12884-016-0916-x PMC4890507 27251052

[42] Lal S, Donnelly C, Shin J. Digital Storytelling: An Innovative Tool for Practice, Education, and Research. Occup Ther Health Care. 2015 1 2;29(1):54–62. 10.3109/07380577.2014.958888 25338054

[43] Njeru JW, Patten CA, Hanza MMK, Brockman TA, Ridgeway JL, Weis JA, et al. Stories for change: development of a diabetes digital storytelling intervention for refugees and immigrants to minnesota using qualitative methods. BMC Public Health [Internet]. 2015 Dec [cited 2017 Nov 6];15(1). Available from: http://www.biomedcentral.com/1471-2458/15/1311 10.1186/s12889-015-2628-y PMC4696160 26715465

[44] Mullany BC. Barriers to and attitudes towards promoting husbands’ involvement in maternal health in Katmandu, Nepal. Soc Sci Med. 2006 6;62(11):2798–809. 10.1016/j.socscimed.2005.11.013 16376007

[45] Mullany BC, Becker S, Hindin M. The impact of including husbands in antenatal health education services on maternal health practices in urban Nepal: results from a randomized controlled trial. Health Educ Res. 2006 7 19;22(2):166–76. 10.1093/her/cyl060 16855015

[46] Dudgeon MR, Inhorn MC. Men’s influences on women’s reproductive health: medical anthropological perspectives. Soc Sci Med. 2004 10;59(7):1379–95. 10.1016/j.socscimed.2003.11.035 15246168

